# User- and Message-Level Correlates of Endorsement and Engagement for HIV-Related Messages on Twitter: Cross-sectional Study

**DOI:** 10.2196/32718

**Published:** 2022-06-17

**Authors:** Jimin Oh, Stephen Bonett, Elissa C Kranzler, Bruno Saconi, Robin Stevens

**Affiliations:** 1 Graduate School of Education University of Pennsylvania Philadelphia, PA United States; 2 School of Nursing University of Pennsylvania Philadelphia, PA United States; 3 Fors Marsh Group Arlington, VA United States; 4 Annenberg School for Communication and Journalism University of Southern California Los Angeles, CA United States

**Keywords:** HIV prevention, social media, public health, young adults, LASSO, HIV, Twitter, digital health

## Abstract

**Background:**

Youth and young adults continue to experience high rates of HIV and are also frequent users of social media. Social media platforms such as Twitter can bolster efforts to promote HIV prevention for these individuals, and while HIV-related messages exist on Twitter, little is known about the impact or reach of these messages for this population.

**Objective:**

This study aims to address this gap in the literature by identifying user and message characteristics that are associated with tweet endorsement (favorited) and engagement (retweeted) among youth and young men (aged 13-24 years).

**Methods:**

In a secondary analysis of data from a study of HIV-related messages posted by young men on Twitter, we used model selection techniques to examine user and tweet-level factors associated with tweet endorsement and engagement.

**Results:**

Tweets from personal user accounts garnered greater endorsement and engagement than tweets from institutional users (aOR 3.27, 95% CI 2.75-3.89; *P*<.001). High follower count was associated with increased endorsement and engagement (aOR 1.05, 95% CI 1.04-1.06; *P*<.001); tweets that discussed STIs garnered lower endorsement and engagement (aOR 0.59, 95% CI 0.47-1.74; *P*<.001).

**Conclusions:**

Findings suggest practitioners should partner with youth to design and disseminate HIV prevention messages on social media, incorporate content that resonates with youth audiences, and work to challenge stigma and foster social norms conducive to open conversation about sex, sexuality, and health.

## Introduction

Despite advances in prevention, the incidence of HIV among youth and young adults in the United States is a continued public health concern. From 2010-2016, adolescents and young adults experienced the highest rates of HIV infection relative to other age groups, with estimates suggesting that the number of individuals living with undiagnosed HIV infection is disproportionately greater within these populations [1]. By the end of 2016, an estimated 50,900 youth were living with HIV [2], yet nearly half (44%) were unaware of their HIV status [3]. These estimates are bolstered by findings that youth and young adults achieve low rates of HIV testing [4]. Moreover, youth and young adults are the least likely of any age group to be linked to HIV care once diagnosed [3] and face unique challenges related to accessing preventative health services [5]. The Ending the HIV Epidemic in the United States initiative highlights the need to expand HIV testing and strengthen linkage to treatment and prevention for populations highly impacted by HIV, including youth and young adults [6].

Social media platforms present unique opportunities for influencing health beliefs and behaviors among users. Such platforms are exceptionally popular among youth and young adult populations; more than 90% of young adults (aged 18-29 years) report having ever used at least one social media platform or messaging app, such as YouTube, Facebook, or Instagram [7], and in recent years, a third or more of teens and young adults reported Twitter use [8]. Young people use Twitter to both engage in conversation within established social networks and communicate with larger audiences [9]. In particular, there is evidence that young people use Twitter as a platform for discussing topics related to sex and health [10-12], creating opportunities for sharing resources and information.

There is substantial evidence that social media use among youth correlates with health outcomes; this research demonstrates both positive and negative health effects among media users [13]. Exposure to alcohol and smoking-related content on social media is correlated with greater self-reported use of alcohol and tobacco products [14,15], highlighting the negative repercussions of media use. However, research has also shown that exposure to sexual health messages on social media is associated with sexual risk reduction behaviors [16], nutrition behavior interventions using social media are linked to increased fruit and vegetable consumption [17], and use of social networking sites for sexual minority youth are associated with positive mental health outcomes.

Media discourse surrounding health topics can play an instrumental role in health-relevant beliefs and behaviors. The dissemination of health-relevant information, during routine exposure to mass media or through purposeful intervention, has been shown to influence health outcomes across a range of behaviors [18]. More specifically, these effects are evident in the domain of HIV/AIDS-related behavior, with evidence that exposure to HIV prevention campaigns through mass media leads to increases in HIV knowledge and greater use of condoms [19]. Social media can fill a similar role in the dissemination of health-related messages, and there is emerging evidence of the impact of social media on HIV-related outcomes [20,21]. Media effects are contingent on message exposure [22], without which audiences cannot receive and process message content. Theories of communication suggest that in addition to message content features, the characteristics of a message source (eg, sender) can influence the extent to which audiences attend to and engage with the message [22], a prerequisite for persuasion and ultimate behavior change [23,24]. Thus, message-consistent outcomes are linked with the extent to which individuals are exposed to a given message and the distinct features of the message source and content.

Previous research suggests that characteristics of message content on social media platforms are related to engagement with health-related messages, including HIV prevention messages [25-27]. This research has suggested that messages with practical information and supportive messages tend to garner greater engagement. The impact of messenger, or message source, on engagement with health messages has also been explored. One study found that messages originating from health-related organizations garnered greater engagement compared to messages from individuals, while messages from non–health-related organized garnered less engagement [25]. Another study found that while health experts were active in producing HIV-related content on Twitter, engagement with these messages was greatest when retweeted by a non–health expert celebrity [28]. Despite the growing interest in the role of social media in health messaging, little research has examined the characteristics of HIV-related social media messages as they relate to youth engagement with such media. To address this gap in the literature, this study aims to explore how user-level characteristics (eg, age, user type, friend count, and follower count) and tweet-level characteristics (eg, format, timing, geolocation, and content) are associated with tweet engagement with and endorsement of Twitter messages posted by adolescent and young adult men in the United States.

## Methods

### Data Description

This study is an expanded analysis of data collected as part of Virus 2 Viral, a study of Twitter message content among young men in the United States [20]. For the Virus 2 Viral study, researchers collected a random sample of tweets from the Twitter fire hose application programming interface (API) posted between January 1, 2016, and December 31, 2016. They filtered this sample to include only users of predicted male gender and predicted age 13 to 24 years (N=336,000 users) using established procedures [29]. For this study, we then expanded the original set of tweets by collecting full timelines (ie, the entire collection of tweets posted by a given user from 2009 to 2017) for those users identified in Virus 2 Viral. The subsequent procedures used to produce the final dataset mirror those described by Stevens et al [20], using this expanded set of tweets. We briefly describe these procedures below.

The initial corpus of tweets was then subset to include only those with HIV-relevant content. HIV-relevant content was identified using a keyword list of HIV-related terms (eg, terms related to HIV, AIDS, HIV testing, condoms, multiple sexual partners, sexually transmitted infections [STIs], sexual risk behavior, and preexposure prophylaxis [PrEP]), developed in partnership with youth researchers. This process generated a dataset of 24,388 tweets that had been posted between 2009 and 2017 and were grouped into 3 broad categories: HIV prevention-specific tweets (n=5057), general sex-related tweets (n=19,319), and risk behavior–promoting tweets (n=12). To retain tweets most relevant to HIV risk and prevention while reducing this data set to a more manageable size, we included the full sample of prevention-related tweets and risk behavior–promoting tweets and a random sample of general sex-related tweets (3091/19,319, 16.0%). This yielded a final data set of 8160 tweets from 1541 unique users that were then coded by a team of 4 research assistants (intraclass correlation coefficient at .80 or higher on all constructs) for message content and used for analysis. User type was determined based on a manual review of the user profile and recent postings of each user in the data set by a member of the research team and was recorded as either individual (eg, a personal account of an individual) or institutional (eg, public health agencies, social service organizations, or advocacy groups). User types that were ambiguous or could otherwise not be determined by the researcher were recorded as missing and were removed from the data set (n=150). The final analytic sample included 8010 tweets from 1499 unique users. A full description of the methods used for the parent study has been published elsewhere [20].

### Ethics Approval

The University of Pennsylvania institutional review board reviewed this study and designated it exempt because the study (protocol #827833) does not meet the definition of human subject research.

### Measure

#### Endorsement and Engagement

Two different binary variables were used to measure the outcomes of tweet endorsement and engagement. A tweet was classified as endorsed if it received at least 1 favorite from another user (1=endorsement, 0=no endorsement) and as engaged if it was retweeted at least once (1=engagement, 0=no engagement).

#### User Characteristics

Number of friends and followers were extracted for each user from the API. Predicted age was estimated using a previously validated machine learning algorithm that predicts user age from characteristics of that user’s messages [29]. User type, determined by manual review of the user profile as described above, was recorded as either individual or institutional.

#### Tweet Characteristics

Tweet language was extracted directly from the API and was coded as a binary variable (1=English, 0=other language). Time of tweet posting was collapsed into 3 categories: daytime for tweets posted between 9 AM and 5 PM EST, evening for tweets posted between 5 PM and midnight EST, and night for tweets posted between midnight and 9 AM EST. The geographic location from which a tweet was posted was measured using tweet-specific latitude/longitude coordinates when available and the self-reported location information in Twitter user profiles otherwise. Tweet locations were then collapsed into a variable to represent region, corresponding with the 4 US Census regions (Northeast, Midwest, South, and West). A tweet was identified as a reply if it was directed at another user using the “@user” syntax (1=reply, 0=not reply). Tweet length was calculated based on the number of characters in the tweet, including “@user” syntax, if present.

#### Tweet Content

The content of a tweet was qualitatively coded by 4 research assistants and consisted of 19 nonexclusive binary variables corresponding to various aspects of the tweet’s content. These categories are anti–risk-taking; condoms; HIV testing; HIV/AIDS; humor; lesbian, gay, bisexual, transgender, or queer; misinformation; modeling; multiple partners; norms; PrEP; pro–risk-taking; research, education, news; stigma; STIs; substance use; transactional sex; unprotected sex; and unrelated sexual content. Full details of the procedures used in the parent study for coding tweet content have been published elsewhere [20].

### Statistical Analysis

A series of logistic regression models were estimated to assess the influence of user-level and tweet-level characteristics on 2 discrete response variables: endorsement and engagement. We used least absolute shrinkage and selection operator (LASSO) as a model building technique. LASSO is a form of penalized regression that forces the regression coefficients of less important variables to zero, yielding models that have fewer variables and higher predictive accuracy [30].

As LASSO regression coefficients are biased and cannot be easily interpreted, we used an extension of this technique known as relaxed LASSO, which sequentially combines the LASSO method for initial model selection with multiple logistic regression for nonpenalized coefficient estimation [31]. Therefore, separate multiple logistic regression models were built for each outcome using the LASSO-selected variables. Final model selection was performed using a backward elimination procedure that only retained predictors statistically significant at the level of .05. From the final multiple logistic models, we estimated adjusted odds ratios (aORs) of predictors of interests while controlling for the effects of covariates. Statistical significance was assessed using *P* values from the Wald chi-square test. All analyses were conducted using the glmnet package [32] in R statistical software (R Foundation for Statistical Computing).

**Figure 1:**
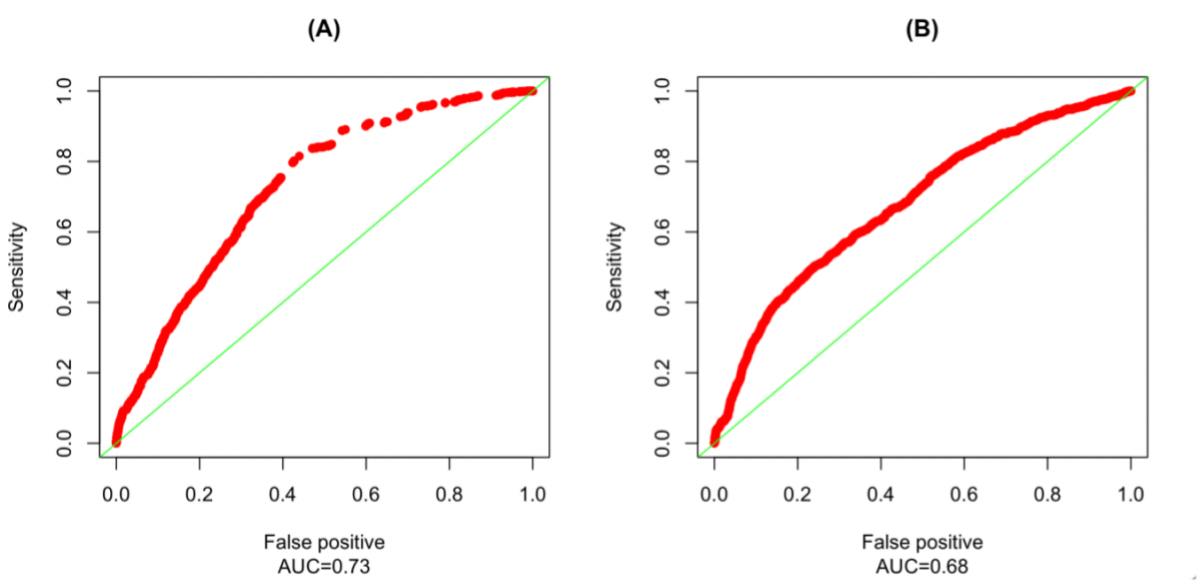
Receiver operating curve and area under the curve for models predicting tweet endorsement (A) and engagement (B).

Finally, to evaluate the overall prediction accuracy of models, we plotted receiver operating curves (ROCs) and calculated the area under the curve (AUCs) [33]. The ROCs, presented in Figure 1, display the relationship between the false positive rate (the proportion of tweets incorrectly classified as endorsed or engaged) and true positive rate (the proportion of tweets correctly classified as endorsed or engaged; also known as sensitivity) of the classifier for all possible thresholds [34], with higher AUC values indicating better predictive power of the model. In other words, each point on the ROC curves indicates the false positive rate and true positive rate of the classifier at a given threshold. ROC curves and AUC are convenient tools to evaluate the performance (accuracy) of the classifier [34]. If the ROC curves were plotted close to the top left corner, this would indicate that the model was able to correctly classify endorsed or engaged tweets with any thresholds at a low false positive rate (AUC would be close to 1). Conversely, if the model could not accurately predict tweet endorsement or engagement (effectively generating random predictions), the ROC curve would be a diagonal line (ie, AUC=0.5).

## Results

### User and Tweet Descriptive Statistics

Table 1 summarizes the descriptive statistics for user and tweet characteristics in the study sample. The mean predicted age of users was 18.72 (SD 3.08) years, with approximately half (4096/8010, 51.1%) identified as institutional users. Number of friends and number of followers were positively skewed. The median number of friends was 435 (IQR 273-800), compared with a mean of 822. The number of followers showed similar patterns, with a median of 591, IQR of 241 to 1179, and mean of 2005 followers. Although the mean number of followers was 2005, most tweets (6008/8010, 75.0%) came from users with fewer than 1179 followers. This difference was due to a small number of users with extremely high numbers of followers. Over half of all tweets (4411/8010, 55.1%) were posted during the daytime, while 26.8% (2146/8010) were posted in the evening and 18.1% (1453/8010) were posted at night. The average tweet length was 94 (SD 31.88) characters with a slight skewness toward longer messages. About 12.0% (959/8010) of tweets were categorized as replies to other users. With respect to tweet content, the most common message categories were HIV/AIDS (4438/8010, 55.4%); research, education, and news (3667/8010, 45.8%); unrelated sexual content (2314/8010, 28.9%); and anti–risk-taking (1208/8010, 15.1%); see Multimedia Appendix 1 for the frequency of each message category. Out of the tweets in the sample, 25.6% (2049/8010) were endorsed and 18.0% (1438/8010) garnered engagement.

**Table 1:** Descriptive statistics for user-level and message-level characteristics (n=8010).

		Value
**Institution, n (%)**		
	Yes	4096 (51.14)
	No	3914 (48.86)
**Location of post, n (%)**		
	Midwest	663 (8.28)
	Northeast	2962 (36.98)
	South	2014 (25.14)
	West	2371 (29.60)
**Message language, n (%)**		
	English	7976 (99.58)
	Not English	34 (0.42)
**Reply, n (%)**		
	Yes	959 (11.97)
	No	7051 (88.03)
**Time of post, n (%)**		
	Daytime (9 AM to 5 PM)	4411 (55.07)
	Evening (5 PM to midnight)	2146 (26.79)
	Night (midnight to 9 AM)	1453 (18.14)
**Year of post, n (%)**		
	2009	30 (0.37)
	2010	6 (0.07)
	2011	62 (0.77)
	2012	62 (0.77)
	2013	158 (1.97)
	2014	346 (4.32)
	2015	1174 (14.66)
	2016	2472 (30.86)
	2017	3700 (46.19)
**Endorsement, n (%)**		
	Yes	2049 (25.58)
	No	5961 (74.42)
**Engagement, n (%)**		
	Yes	1438 (17.95)
	No	6572 (82.05)
Age^a^ (years), median (IQR)		18.72 (17.13-21.64)
Follower count, median (IQR)		591 (241-1179)
Friend count, median (IQR)		435 (273-800)
Message length, median (IQR)		94 (71-121)

^a^Age is a predicted age, computed based on tweet and user characteristics using machine learning algorithms developed by Sap et al [29].

### Factors Associated With Tweet Endorsement and Engagement

For each outcome of interest (tweet endorsement and tweet engagement), we estimated logistic regression models using LASSO-selected predictors and assessed overall model performance by plotting ROCs and measuring AUCs. We note that the initial model included all the variables (excluding the outcomes) listed in Table 1 and Multimedia Appendix 1 as predictors.

#### Endorsement

The final model (score test χ^2^_6_: 884.65) for the outcome of tweet endorsement was a 6-variable model, which included the following predictors: number of followers; region; year of tweet posted; user type; STI message content; and research, education, and news message content. As demonstrated in Figure 1, this model had an AUC of 0.73, suggesting acceptable performance [35].

As shown in Table 2, both user-level and tweet-level characteristics were significantly associated with tweet endorsement. With respect to user-level characteristics, the odds of a tweet being endorsed were 3.27 higher for tweets from personal user accounts compared with institutional users (aOR 3.27, 95% CI 2.75-3.89; *P*<.001), and each additional 100 followers that a user had was associated with a 0.53% increase in the odds that their tweet was endorsed (aOR 1.01, 95% CI 1.00-1.01; *P*<.001). User region was also significantly associated with endorsement. Regarding tweet-level characteristics, tweets discussing specific STIs had 41% lower odds of being endorsed, relative to tweets that did not discuss STIs (aOR 0.59, 95% CI 0.47-1.74; *P*<.001). Additionally, tweets that included discussion of research, education, or news related to HIV had 23% lower odds of being endorsed, compared with tweets that discussed HIV in a different context (aOR 0.77, 95% CI 0.65-0.92; *P*<.001). Year of posting was also significantly associated with endorsement.

**Table 2:** Summary of logistic regression analysis for variables predicting endorsement and engagement of Twitter users (n=8010).

Predictor				Endorsement, aOR^a^ (95% CI)		Engagement, aOR (95% CI)
**User level**						
	Age			—^b^		0.92 (0.90-0.94)
	Follower count (100 counts)			1.01 (1.00-1.01)		1.01 (1.00-1.01)
	Personal user count			3.27 (2.75-3.89)		1.77 (1.52-2.05)
**Tweet level**						
	**Region^c^**					
		Northeast	1.46 (1.31-1.99)		1.69 (1.32-2.15)	
		South	0.85 (0.82-1.25)		1.16 (0.91-1.48)	
		West	1.06 (0.71-1.08)		0.68 (0.53-0.88)	
	**Time^d^**					
		Night	—		1.08 (0.90-1.31)	
		Daytime	—		1.36 (1.17-1.59)	
	Message length (10 words)			—		1.04 (1.02-1.06)
	Reply			—		0.45 (0.36-0.57)
	Year			1.30 (1.23-1.38)		—
	Message: norm			—		1.62 (1.15-2.29)
	Message: research, education, news			0.77 (0.65-0.92)		—
	Message: STI			0.59 (0.47-0.74)		0.61 (0.47-0.78)

^a^aOR: adjusted odds ratio.

^b^Not applicable.

^c^Reference group: Midwest.

^d^Reference group: evening.

#### Engagement

The final model (score test χ^2^_9_: 404.89) for the outcome of tweet engagement included the following 9 predictors: predicted user age, number of followers, user type, tweet length, reply tweet (@user), time of post, region, norms message content, and STI message content. As demonstrated in Figure 1, the 9-variable model showed an AUC of 0.68, performing slightly below the acceptable threshold of 0.70 [35].

As shown in Table 2, both user-level and tweet-level characteristics were significantly associated with tweet engagement. For each additional year in the user’s predicted age, the odds of a tweet garnering engagement decreased by 8% (aOR 0.92, 95% CI 0.90-0.94; *P*<.001). Additionally, tweets from personal user accounts (compared with institutional users) had 77% greater odds of garnering engagement (aOR 1.77, 95% CI 1.52-2.05; *P*<.001). Each additional 100 followers was associated with a 0.51% increase in the odds of a tweet garnering engagement (aOR 1.01, 95% CI 1.00-1.01; *P*<.001). Tweets that were replies (@user) were 55% less likely to garner engagement from other users (aOR 0.45, 95% CI 0.36-0.57; *P*<.001). User region was also significantly associated with engagement. Regarding tweet-level characteristics, tweets that discussed STIs had 39% lower odds of garnering engagement compared to tweets that did not discuss STIs (aOR 0.61, 95% CI 0.47-0.78; *P*<.001). Tweets that included discussion of social norms were 62% more likely to garner engagement compared with tweets that did not discuss social norms (aOR 1.62, 95% CI 1.15-2.29; *P*<.001). Tweet length and time of posting were also significantly associated with engagement.

## Discussion

### Principal Findings

This study was designed to assess the relationships between user-level and tweet-level characteristics and endorsement and engagement of tweets related to HIV risk and prevention posted by young men. Our analysis demonstrated that characteristics both of users and of the tweets themselves were associated with tweet endorsement and engagement. Given that fostering active interaction with media content around HIV prevention is a critical component of a public health social media strategy [36], these results have important implications for HIV prevention efforts.

We found that tweets from personal accounts were 3 times more likely to be endorsed, and 75% more likely to garner engagement, when compared with institutional users. This finding suggests that message source is an important factor in how HIV-related tweets are received and that HIV-relevant messages from institutional users may not resonate as strongly with youth. Previous research has shown that while institutional sources of online HIV information may be perceived as more credible, the experiences of peers may be more influential in shaping attitudes and self-efficacy to change behaviors [37]. Public health messaging efforts around HIV prevention should acknowledge these findings when considering how to use resources related to online communication; using institutional accounts to post messages to social media platforms may not result in meaningful engagement from youth. Thus, promoting peer-to-peer discussions of HIV-related topics through social media interventions may have greater potential to influence the attitudes and behaviors of youth [38]. However, it is important to note that although institutional tweets were not often retweeted or favorited, it is possible that they were still read by many users and the information was communicated as intended.

Results demonstrated that users with many followers were more likely to garner tweet endorsement and engagement relative to users with fewer followers; each additional 100 followers were associated with a 0.5% increase in the odds of both endorsement and engagement. This is not a surprising finding, given that having more followers increases one’s opportunity for tweet exposure, thereby increasing the likelihood that a given tweet is endorsed or elicits engagement. We did not find any association between users’ number of friends and endorsement or engagement, which suggests that having a robust following on Twitter may be more important than being highly connected to other users through friendship. Users with large followings may be celebrities or social media influencers, or simply perceived as such, and their position of influence could be leveraged to increase visibility of HIV prevention messages. However, considering the highly skewed distribution of followers in this data set, the relationship between the odds of endorsement or engagement and the follower count may not tell the whole story. Users may be more likely to engage with the messages from microinfluencers (eg, an influential user with fewer than 10,000 followers) than from celebrity influencers (eg, an influential user with more than 10,000 followers) due to feeling a closer sense of connection with these microinfluencers [39]; however, additional research on these relationships is warranted. These distinctions aside, influencers are well positioned to reach a large audience on Twitter and could be an important component of public health campaigns or other messaging efforts that use social media to engage with young people [40,41].

The findings from this study have implications for the implementation of popular opinion leader (POL) interventions. POL interventions aim to identify, enlist, and train key opinion leaders in a community to promote health behaviors and challenge risky social norms [42]. These leaders act as early adopters of behavior change and can serve as models and supports for peers who are considering making similar changes. Our results demonstrate that, in addition to such characteristics as the quality and originality of message content, users on social media with large numbers of followers may be positioned to garner significant engagement with their messages, thus making them good candidates as opinion leaders [43]. Future intervention development should seek ways to integrate the principles of POL into interventions related to HIV prevention through online social media.

Findings also demonstrated that the content of messages on Twitter was related to tweet endorsement and engagement. Tweets that mentioned STIs garnered decreased endorsement and decreased engagement, and tweets that were primarily focused on research, education, or news showed lower levels of endorsement. However, tweets that reflected social norms (an opinion about how oneself or others behave or should behave) garnered higher levels of engagement, suggesting that young people are eager to participate in conversations about the perceived behaviors of peers or evaluations of those behaviors. These results have important implications for efforts to develop health communication tools for HIV prevention. Stigma surrounding HIV and STIs may stifle conversations about sexual health, in light of evidence that young people tend to distance themselves from direct discussion of these issues in settings that are not sufficiently anonymous or confidential [44]. Furthermore, tweets that highlight research, education, or news about sexual health may not resonate with young people, leading to low rates of endorsement. Health communication around HIV prevention must balance an acknowledgment of this stigma without further reinforcing it. Rather than avoid direct discussion of issues related to HIV prevention, public health educational efforts should embed these discussions in the larger context of sex and sexuality and connect these discussions to the social realities that young people live in (ie, acknowledging and/or challenging social norms).

Additional characteristics of messages were found to be associated with endorsement, engagement, or both. Users with greater predicted age showed lower odds of garnering engagement, which may reflect differences in platform use between adolescents and young adults. Variations in endorsement and engagement were seen by geographic region, with messages originating from the Northeast of the United States receiving the greatest levels of endorsement and engagement, mirroring the geographic distribution of Twitter activity that has been seen in previous studies [45]. Longer tweets received greater engagement, a finding that has been described in previous studies [28]. Previous studies have shown that engagement with messages on Twitter varies across the day and according to message content [46]. The variation in message engagement seen in our study, where engagement was highest for messages posted during the day and lowest during the evening, highlights the need to consider time of posting for public health messages. Replies garnered low engagement in our study, suggesting that dialogues between users about HIV do not stimulate engagement from young people. Finally messages posted during later years in the study received greater endorsement, likely reflecting a growth in the popularity of the platform over the study period.

Public health efforts to incorporate social media messaging into HIV prevention approaches will require novel strategies around message creation, delivery, and evaluation. The use of language and style that leverages the cultural elements of social media, such as incorporating memes and sharable elements into message content, may resonate more effectively with young people than appeals based solely on facts and knowledge [41]. Future research should aim to collect additional information about tweets, including qualitative codes related to themes beyond HIV prevention (eg, presence of a meme, celebrity reference), that may correlate more strongly with tweet engagement and endorsement. Furthermore, the use of POL techniques could help to overcome and challenge stigma around sexual health, allowing information about HIV prevention to be visible on social media platforms.

### Limitations

This study is subject to several notable limitations. First, our outcomes of tweet endorsement and engagement capture active interactions with social media content, not passive exposure to tweet content. Young people may be hesitant to endorse messages related to sex and sexual health because of stigma or embarrassment but may still be reading these messages anonymously [47]. However, data on tweet views are difficult to obtain, and research may be limited to measures of endorsement and engagement similar to ours. Second, there were several users who contributed a very large number of tweets (eg, one user accounted for 949 tweets) in this data set, raising concerns about the independence of observations. While capturing highly active and widely followed Twitter accounts is important to this line of work, future analyses should consider models that account for clustering of errors at the user level. Third, it is important to note that our models for tweet endorsement and tweet engagement showed only a modest capacity to discriminate between tweets that evinced the outcome and tweets that did not (acceptable discrimination for endorsement and slightly less than acceptable discrimination for engagement). While our study suggests that user and tweet-level characteristics have measurable associations with tweet endorsement and engagement, further work is needed to identify additional characteristics of users and tweets that might strengthen predictive modeling for endorsement and engagement with HIV-related messages on Twitter. Finally, it should also be noted that messages analyzed in this study were limited to Twitter messages geolocated to the United States. The patterns seen in our study may not be generalizable to social media messages on other platforms or in other countries.

### Conclusions

The widespread use of social media platforms among young people offers new opportunities for communication around HIV prevention. Conversations about sex and sexual health are widespread across these platforms, providing an opportunity for public health messaging to play a role in these conversations. Efforts to engage with young people on these sensitive and often stigmatized topics will require innovative strategies to foster meaningful connection with HIV prevention messages. Public health practitioners should partner with young people to design and disseminate these messages, incorporate content that resonates with youth audiences, and work to challenge stigma and foster social norms conducive to open and honest conversation about sex, sexuality, and health.

## Appendix

Multimedia Appendix 1 Qualitatively coded message frequency table (N=8010).

## Abbreviations

aOR: adjusted odds ratio
API: application programming interface
AUC: area under the ROC curve
LASSO: least absolute shrinkage and selection operator
POL: popular opinion leader
PrEP: preexposure prophylaxis
ROC: receive operating curve
STI: sexually transmitted infection
