# Supplementary material for: User- and Message-Level Correlates of Endorsement and Engagement for HIV-Related Messages on Twitter: Cross-sectional Study
Source: JMIR Public Health Surveill. 2022 Jun 17;8(6):e32718. doi: 10.2196/32718 (PMC9250060; doi:10.2196/32718)
Supplement: Multimedia Appendix 1 [file publichealth_v8i6e32718_app1.docx]

**Multimedia Appendix 1.** Supplemental material.

Table S1. Qualitatively coded message frequency table (*n* = 8010).

|  | **Frequency (%)** |
| --- | --- |
| **Anti risk taking**  Yes  No | 1208 (15.08%)  6802 (84.92%) |
| **Condoms**  Yes  No | 199 (2.48%)  7811 (97.52%) |
| **HIV testing**  Yes  No | 979 (12.22%)  7031 (87.78%) |
| **HIV AIDS**  Yes  No | 4438 (55.41%)  3572 (44.59%) |
| **Humor, fear, clapback**  Yes  No | 140 (1.75%)  7870 (98.25%) |
| **LGBTQ**  Yes  No | 319 (4.00%)  7691(96.00%) |
| **Look at this one**  Yes  No | 60 (0.75%)  7950 (99.25%) |
| **Misinformation**  Yes  No | 20 (0.25%)  7990 (99.75%) |
| **Modeling**  Yes  No | 21 (0.26%)  7989 (99.74%) |
| **Multiple partners**  Yes  No | 118 (1.47%)  7892 (98.53%) |
| **Music lyrics**  Yes  No | 49 (0.61%)  7961 (99.39%) |
| **Norm**  Yes  No | 188 (2.35%)  7822 (97.65%) |
| **Pharmaceutical marketing**  Yes  No | 4 (0.05%)  8006 (99.95%) |
| **Porn**  Yes  No | 23 (0.29%)  7987 (99.71%) |
| **PrEP**  Yes  No | 229 (2.86%)  7781 (97.14%) |
| **Pro risk taking**  Yes  No | 68 (0.85%)  7942 (99.15%) |
| **Research education news**  Yes  No | 3667 (45.78%)  4343 (54.22%) |
| **Shaming and stigma**  Yes  No | 87 (1.09%)  7923 (98.91%) |
| **STI**  Yes  No | 630 (7.87%)  7380 (92.13%) |
| **Substance use**  Yes  No | 206 (2.57%)  7904 (97.43%) |
| **Transactional sex**  Yes  No | 52 (0.65%)  7958 (99.35%) |
| **Unprotected sex**  Yes  No | 87 (1.09%)  7923 (98.91%) |
| **Unrelated sexual content**  Yes  No | 2314 (28.89%)  5696 (71.11%) |
| **Unrelated to sex**  Yes  No | 855 (10.67%)  7155 (89.33%) |
